# Experience and lessons learned relating to investigational product supply in the design and delivery of a paediatric investigator-initiated clinical trial

**DOI:** 10.1016/j.conctc.2025.101517

**Published:** 2025-06-30

**Authors:** Mandy Wan, Mark A. Turner, Gilles Cambonie, Ruth Kemper, Naouel Bouafia, Lea Levoyer, Alpha Diallo, Mikko Hallman, Jean-Christophe Rozé

**Affiliations:** a Guy's and St Thomas' NHS Foundation Trust, Evelina London Children's Hospital, Pharmacy Department, London, UK; b Institute of Pharmaceutical Science, King's College London, London, UK; c Institute of Life Course and Medical Sciences, University of Liverpool, Liverpool, UK; d Conect4children Stichting, Utrecht, Netherlands; e Department of Neonatal Medicine and Pediatric Intensive Care, Arnaud de Villeneuve Hospital, Montpellier University Hospital, Montpellier, France; f Pathogenesis and Control of Chronic Infection, INSERM, University of Montpellier, Montpellier, France; g European Foundation for the Care of Newborn Infants, EFCNI, Munich, Germany; h Clinical Trial Safety and Public Health, ANRS|Emerging Infectious Diseases, Paris, France; i Clinical Research Safety Department, INSERM, Paris, France; j Department of Pediatrics and Adolescent Medicine, Oulu University Hospital, and Research Unit of Clinical Medicine and MRC Oulu, University of Oulu, Oulu, Finland; k Department of Neonatalogy, Nantes University Hospital, Nantes, France; l Centre d'Investigation Clinique CIC1413, INSERM-Nantes University Hospital, Nantes, France

**Keywords:** Investigational drugs, Investigator initiated clinical trials

## Abstract

The management of investigational product (IP) supply is a complex endeavour when designing and delivering clinical trials. In contrast to industry-sponsored trials where IP supplies are coordinated by teams of specialists working together throughout the entire supply chain, investigator initiated clinical trials often face IP-related challenges that can result in substantial trial delays, higher costs, and even early termination of the trial. Despite the challenges faced by investigators, there has been relatively few discussions on this topic in the literature. In this short communication, we describe our experiences and the lessons learned in managing IP supply during the design and execution of a multinational paediatric investigator initiated clinical trial. These experiences are shared to provide researchers with tools and strategies to improve the future implementation of investigator-initiated clinical trials.

## 1 Introduction

The historical lack of clinical trials involving infants and children has led to widespread off-label drug use in paediatric practice. A systematic review from 2018 reported considerable variability in off-label drug use across countries and healthcare settings, with rates ranging from 3.2 % to 95 % [1]. As off-label drug use is generally not supported by the same level of safety and effectiveness data as medicines authorised for paediatric use, it poses inherent risks of adverse drug reactions and suboptimal clinical effectiveness. While there is still much to be learned about the use of these medicines in children, there is a noticeable lack of industry incentive to invest in the development of these off-patent medicines for the paediatric population [2,3]. In this respect, investigator-initiated clinical trials (IITs) play a crucial role in advancing evidence-based paediatric practice.

Conducting IITs requires investigators and non-industry sponsors to manage a wide range of tasks, including regulatory compliance, quality assurance, data management, staff training, and inter-organisational financial activities [2,4]. Although there is a strong acknowledgment of these challenges and the necessity for a supportive research infrastructure to assist investigators and research personnel in executing these trials [5,6], comparatively little attention has been given to the specific challenges associated with the supply of medicines required by trial protocols (investigational products (IP), also known as investigational medicinal products).

Clinical trials pharmacy services, or investigational drug services, as they are known in the United States and Canada, primarily focus on site-level responsibilities related to the proper handling of IP. These hospital or academic pharmacy units do not typically undertake broader coordination roles in multicentre trials, and even fewer are involved in managing IP activities across multinational studies [7]. In industry-sponsored trials, such coordination is typically managed by commercial IP supply companies, where specialised teams manage the full IP supply chain, from sourcing and manufacturing to blinding, packaging and labelling, storage and distribution, all in full compliance with regulatory requirements. By comparison, IITs, which typically operate with fewer resources and specialised expertise, often face IP-related challenges that can result in substantial trial delays, higher costs, and even early termination of the trial [8]. Investigators have identified this area as one that requires significant support [2,4,9], highlighting a relevant gap that could be addressed by paediatric research networks and infrastructure.

The conect4children (c4c) project [https://conect4children.org/] is a paediatric pan-European collaborative clinical trial network comprising academia, speciality networks, and the private sector, aimed at developing services that facilitate the conduct of high-quality clinical trials in babies, children, and young people [10]. It provides a strategic feasibility advice service and an education platform covering all aspects of trial design and delivery for paediatric clinical trials. Here, we describe our experience and the lessons learned in managing IP supply during the design and execution of a multinational paediatric IIT.

### 1.1 TREOCAPA trial

The TREOCAPA trial was one of the non-industry trials conducted under the umbrella of the c4c project in order to assess the viability of the network. It has two parts, Phase II (dose finding open-label) and Phase III. The Phase III part is a randomised, multi-centre, double-blind, stratified, placebo-controlled, two-arm (1:1), superiority trial that was designed to evaluate whether the prophylactic use of paracetamol in extremely premature neonates is safe and effective to close the ductus arteriosus blood vessel and reduces the risk of prematurity associated complications (ClinicalTrials.gov Identifier: NCT04459117). It aimed to recruit 794 premature neonates (23–28 weeks of gestational age), and eligible patients were randomised 1:1 to receive paracetamol or placebo intravenously four times a day for five days, starting within 12 h after birth. The trial was funded by the c4c consortium and sponsored by the Institut National de la Santé Et de la Recherche Médicale (INSERM) in France.

### 1.2 Problem statement

TREOCAPA builds on the work of Härkin et al. (2016) who conducted a randomised, double-blinded trial of paracetamol in a single neonatal intensive care unit (NICU) in Finland [11]. In this single-centre trial, the hospital's routine stock of intravenous paracetamol and sodium chloride 0.45 % solution (as placebo) were used as IPs [11]. Due to visible differences between the commercial paracetamol and sodium chloride products, a separate unblinded team of nurses was required to prepare the IP in a study pharmacy outside of the NICU to ensure that the nurses and doctors caring for the infants remained blinded [11].

Establishing a clear separation of roles between blinded and unblinded study staff at an investigator site presents notable challenges. To minimise the risk of accidental unblinding, unblinded staff are typically pharmacy personnel who operate remotely from the clinical setting and are not directly involved in patient care. However, this arrangement can introduce several limitations, particularly in trials like TREOCAPA, where round-the-clock patient recruitment and frequent dosing make it impractical for pharmacy staff to be involved. Conversely, managing separate blinded and unblinded nursing teams within the same busy clinical environment is practically challenging, and the risk of accidental unblinding is high, even with considerable effort and additional resources.

While Härkin et al. (2016) effectively achieved blinding in their single-centre trial by implementing separate blinded and unblinded nursing teams [11], this approach was deemed unfeasible for TREOCAPA, a trial planned to be conducted in 50 NICUs across 15 European countries. A double-blinded clinical trial of this scale would benefit from a centralised IP supply model. However, for TREOCAPA, there were several blinding options for implementing the centralised IP supply that required careful consideration (Table 1) due to significant variation in pharmaceutical complexity and the potential for associated costs to range from €400,000 to €800,000, or higher, depending on the selected approach.

**Table 1:** Evaluation of different centralised IP supply options in planning the TREOCAPA trial.

Option	Blinding strategy	Considerations
A	Use of bespoke blinding cover/box to conceal the visible differences between commercial paracetamol and sodium chloride 0.9 % or glucose 5 % products	- For this method to be successful, the commercial paracetamol and sodium chloride 0.9 % or glucose 5 % products need to have some degree of similarities in their packaging.
		- Modest cost
B	Repackaging of commercial sodium chloride 0.9 % or glucose 5 % product into a container system that matches the commercial paracetamol product	- Requires the sourcing of container system that matches the commercial paracetamol product.
		- Requires specialist pharmaceutical manufacturer with sterile product capabilities.
		- Moderate cost.
C	Repackaging of commercial paracetamol and sodium chloride 0.9 % or glucose 5 % products into identical container systems	- The repackaging process affects the sterility of the products.
		- The use of different container system requires additional stability testing to determine IP expiration date.
		- Requires specialist pharmaceutical manufacturer with sterile product capabilities.
		- Substantial cost.
D	Bespoke manufacturing of a paracetamol product and a matching placebo in identical container systems	- The paracetamol product requires formulation development.
		- Lengthy stability testing to determine IP expiration date.
		- Requires specialist pharmaceutical manufacturer with sterile product capabilities.
		- Substantial cost.

## 2 Work done

**Fig. 1:**
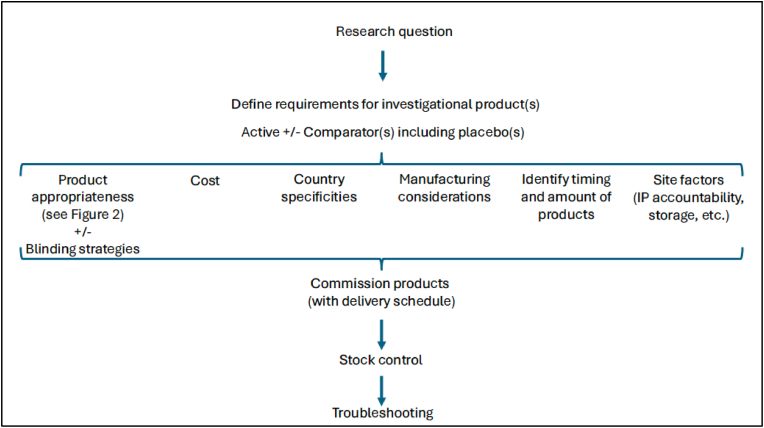
Summary of IP supply considerations.

The c4c consortium, together with its pharmaceutical experts, supported the TREOCAPA team during the early stages of trial design by assessing and designing on all aspects of IP supply, summarised in Fig. 1. The dominant issue was the feasibility of various IP blinding methods, requiring an assessment of the appropriateness of different commercially available paracetamol products for use as IP, and sourcing a matching placebo.

For trials involving off-patent drugs, it is prudent to first evaluate the feasibility of using products that have marketing authorisation and are commercially available as IP. These products offer quality assurance as they are manufactured in compliance with Good Manufacturing Practice (GMP) and meet the regulatory quality standards necessary for clinical trials (note: differences in GMP across countries should be considered). The use of authorised medicinal products as IP eliminates the need for bespoke manufacturing, generally reducing trial setup time and costs. Additionally, it is important to explore all commercially available options, as off-patent drugs are often available in several generic versions that may vary in excipients, colour, shape, or markings, potentially impacting placebo sourcing or manufacturing options. The assessment algorithm shown in Fig. 1 were used to ensure that all clinical and pharmaceutical aspects were comprehensively evaluated for each product.

Moreover, the appropriateness of an authorised medicinal product for use as IP is highly dependent on its packaging. Supplementary Fig. 1 presents examples of the various authorised intravenous paracetamol products available on the market. As illustrated, the different packaging designs pose distinct challenges for placebo manufacturing. Notably, these challenges include sourcing identical packaging for placebo manufacturing, removing or concealing existing labels, and identifying a placebo manufacturer with the relevant capabilities (i.e. a manufacturer may have capability to handle only glass vials of a certain size while another may only have machinery for handling plastic vials).

The optimal solution was identified after an expert in IP supply to paediatric trials spent over 50 h identifying options, assessing clinical and pharmaceutical appropriateness, contacting suppliers, supporting tender documents in procurement, and liaising with the study team. This work was not possible for the clinicians and trial managers in the team.

## 3 The optimal IP supply plan

The trial was conducted using an authorised paracetamol product sourced from France while a well-matched authorised sodium chloride 0.9 % product was imported from the United Kingdom (UK); both products were commercially available as 10 ml plastic ampoules. A pharmaceutical company based in France specialising in clinical trials was contracted to perform the blinding and packaging activities. The two products were rendered indistinguishable after the commercial labels were removed and blinded labels were applied to each ampoule (Supplementary Fig. 2). Ampoules were then packaged into individual patient kits and distributed to participating sites, each uniquely numbered with a kit identifier to enable blinded treatment allocation. Overall, the cost of implementing a centralised IP supply model for the TREOCAPA trial was modest and reduced the risk of accidental unblinding. In comparison, maintaining separate blinded and unblinded nursing teams at each of the 50 sites would have incurred additional staffing costs, conservatively estimated at €10,000 per site annually, amounting to over €1 million for an average two-year recruitment period per site, with actual costs likely higher due to the longer duration of trial recruitment.

### 3.1 Lessons learnt

For TREOCAPA, it is important to note that, although the final IP supply plan was deemed more efficient and cost-effective compared to other options listed in Table 1, it was entirely reliant on the commercial pharmaceutical market and thus vulnerable to supply chain disruptions. In the case of TREOCAPA, the UK's exit from the European Union led to a short-term disruption in the supply of sodium chloride 0.9 % due to importation delay as a result of a change in ownership of the product. Nonetheless, with a proactive approach to IP management and monitoring, this ensured the continuity of IP supply to enable patient recruitment at sites.

The pharmaceutical expertise provided by c4c was essential to TREOCAPA's success, particularly the knowledge and insights related to the international pharmaceutical market, which significantly enhanced the trial's feasibility, reduced the time to open the study, and troubleshoot issues arising during the trial. The pharmaceutical market is complex and highly regulated, with product availability varying from country to country. Not all countries will market a product from a particular manufacturer, and some may market only specific dosages forms or strengths. By leveraging c4c consortium's expertise, TREOCAPA was conducted as a double-blind, placebo-controlled trial without requiring bespoke manufacturing of paracetamol or placebo, nor the need for separate blinded and unblinded site study personnel.

**Fig. 2:**
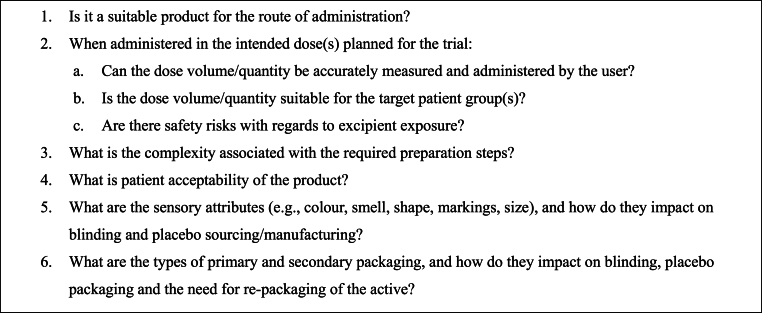
An algorithm for evaluating the appropriateness of an authorised medicinal product for use as an investigational product in a clinical trial.

In this case study, we highlighted the pharmaceutical considerations and challenges associated with coordinating IP supply for a multinational paediatric IIT, and outlined strategies (Fig. 1, Fig. 2) that can inform the broader planning and execution of IITs. In contrast to site-level IP management, which focuses on the local implementation of trial protocols using sponsor-provided IP, the coordination of IP supply across multicentre or multinational trials presents a distinct and more complex set of challenges. These include the integration of clinical and pharmaceutical considerations into trial design, large-scale operational planning, navigation of country-specific regulatory requirements, management of cross-border supply chain and logistics, and accommodation of variability in local infrastructure and site-level practices. The complexity of such coordination demands specialised expertise and operational insight, which may not yet be fully developed within local pharmacy investigational drug services or clinical trials coordinating centres (clinical trials units). Yet, the established site-level expertise of pharmacy teams in IP management and their experience with diverse trial protocols underscore an opportunity to build on this skill set by enhancing their capabilities to support centralised IP supply coordination and embedding their expertise within broader research networks, thereby strengthening the infrastructure necessary for the effective delivery of IITs.

## 4 Conclusion

In this article, we share our learning on coordinating IP supply from the TREOCAPA trial, demonstrating how pharmaceutical expertise within a research network is a crucial aspect to supporting the design and conduct of paediatric IITs for off-patent drugs. By providing a multi-expertise platform, the c4c consortium and other research networks can support researchers through shared learning and facilitate efficient trial implementation which can significantly improve trial delivery and performance [6,10]. As a c4c non-industry trial, the knowledge acquired from TREOCAPA will further inform the strategic feasibility advice services offered by the network, enhancing its contributions to advancing paediatric clinical trials.

## CRediT authorship contribution statement

**Mandy Wan:** Writing – original draft, Conceptualization. **Mark A. Turner:** Writing – review & editing. **Gilles Cambonie:** Writing – review & editing. **Ruth Kemper:** Writing – review & editing. **Naouel Bouafia:** Writing – review & editing. **Lea Levoyer:** Writing – review & editing. **Alpha Diallo:** Writing – review & editing. **Mikko Hallman:** Writing – review & editing, Funding acquisition. **Jean-Christophe Rozé:** Writing – review & editing, Funding acquisition.

## Consent to participate

N/A.

## Disclaimer

The publication reflects the author's view and neither IMI nor the European Union, EFPIA, or any Associated Partners are responsible for any use that may be made of the information contained therein.

## Availability of data and material (data transparency)

N/A.

## Code availability

N/A.

## Ethics approval

N/A.

## Consent for publication

N/A.

## Funding

The conect4children (c4c)-Collaborative Network for European Clinical Trials for Children project has received funding from the 10.13039/501100010767Innovative Medicines Initiative 2 Joint Undertaking under grant agreement No 777389. The Joint Undertaking receives support from the European Union's 10.13039/501100007601Horizon 2020 research and innovation programme and 10.13039/100013322EFPIA.

## Declaration of competing interest

The authors declare the following financial interests/personal relationships which may be considered as potential competing interests:All the authors are associated with the c4c project because their employers are Beneficiaries or Third Parties of the c4c consortium.
