# Supplementary material for: Experience and lessons learned relating to investigational product supply in the design and delivery of a paediatric investigator-initiated clinical trial
Source: Contemp Clin Trials Commun. 2025 Jun 30;46:101517. doi: 10.1016/j.conctc.2025.101517 (PMC12270041; doi:10.1016/j.conctc.2025.101517)
Supplement: Multimedia component 1 [file mmc1.docx]

**Supplementary File**


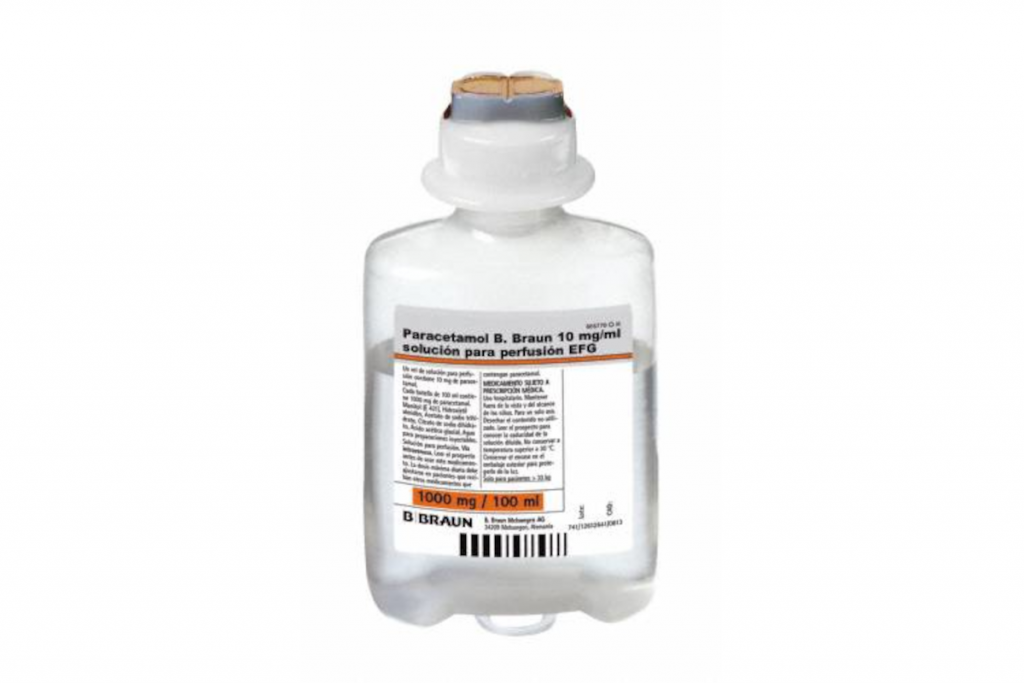

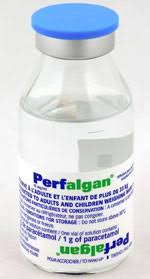

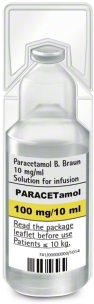

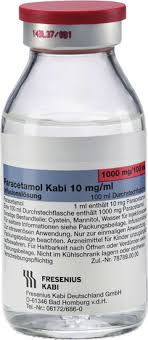

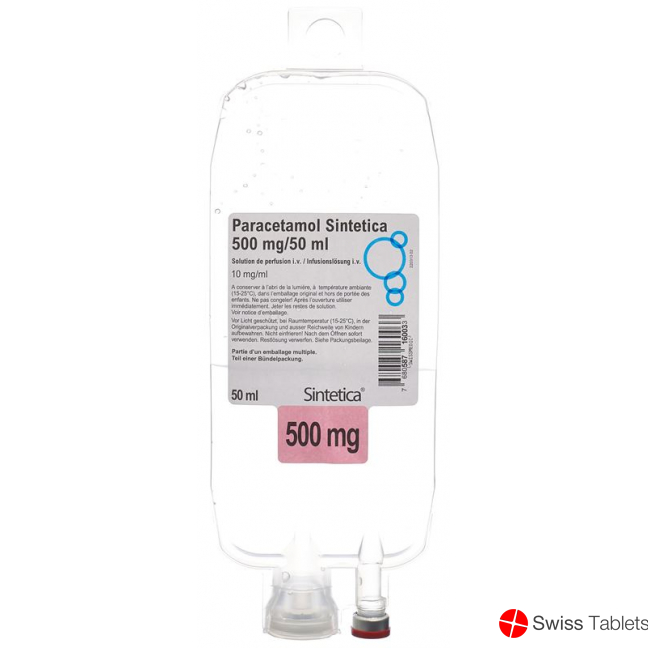


**Supplementary Figure 1: Examples of the different authorised intravenous paracetamol products available on the market**


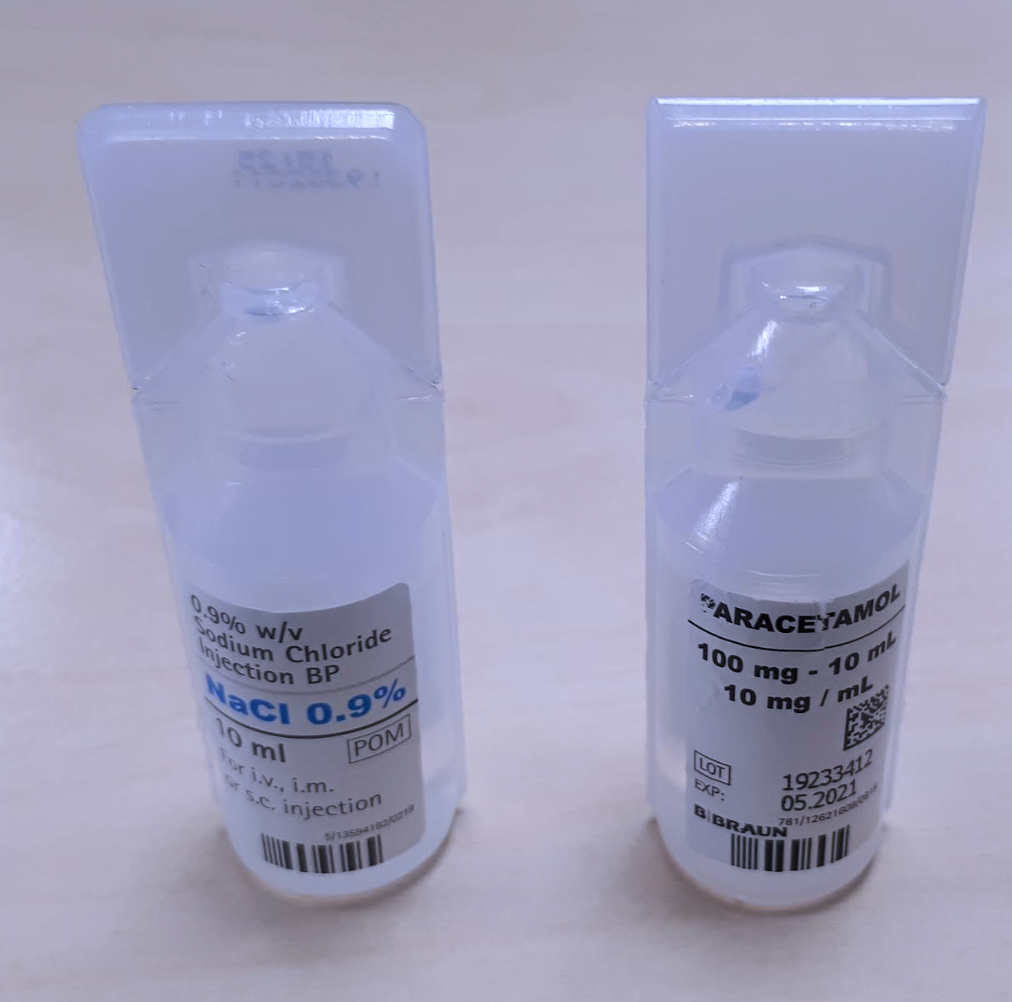

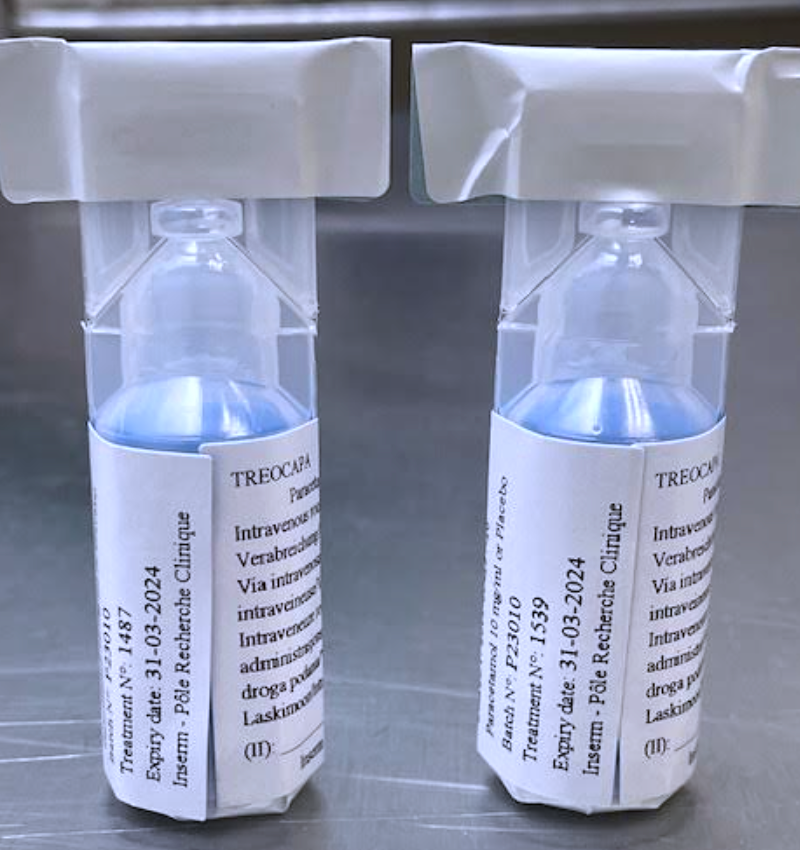


**Supplementary Figure 2: Investigational products used in TREOCAPA trial (Left: before blinding)**
